# Experiences of bereaved family caregivers with shared decision making in palliative cancer treatment: a qualitative interview study

**DOI:** 10.1186/s12904-021-00833-z

**Published:** 2021-09-07

**Authors:** Sanne P. C. van Oosterhout, Daisy J. M. Ermers, Floor K. Ploos van Amstel, Carla M. L. van Herpen, Yvonne Schoon, Marieke Perry, Maartje van Geel, Evelien J. M. Kuip, Yvonne Engels

**Affiliations:** 1 Department of Anesthesiology, Pain and Palliative Medicine, Radboud University Medical Center, Nijmegen, The Netherlands; 2 Department of IQ healthcare, Radboud Institute for Health Sciences, Radboud University Medical Center, Kapittelweg 54 (160), 6525 EP Nijmegen, The Netherlands; 3 Department of Medical Oncology, Radboud University Medical Center, Nijmegen, The Netherlands; 4 Department of Geriatric Medicine, Radboud University Medical Center, Nijmegen, The Netherlands; 5 Department of Primary and Community Care, Radboud University Medical Center, Nijmegen, The Netherlands; 6 Alzheimer Center, Radboud University Medical Center, Nijmegen, The Netherlands; 7 Department of Process Improvement and Innovation, Radboud University Medical Center, Nijmegen, The Netherlands

**Keywords:** Family caregivers, Palliative care, Shared decision making, Cancer, Bereavement, Communication, Qualitative research, Professional-family relations

## Abstract

**Background:**

Patients with incurable cancer face complex medical decisions. Their family caregivers play a prominent role in shared decision making processes, but we lack insights into their experiences. In this study, we explored how bereaved family caregivers experienced the shared decision making process.

**Methods:**

We performed a qualitative interview study with in-depth interviews analysed with inductive content analysis. We used a purposive sample of bereaved family caregivers (*n* = 16) of patients with cancer treated in a tertiary university hospital in the Netherlands.

**Results:**

Four themes were identified: 1. scenarios of decision making, 2. future death of the patient 3. factors influencing choices when making a treatment decision, and 4. preconditions for the decision making process. Most family caregivers deferred decisions to the patient or physician. Talking about the patient’s future death was not preferred by all family caregivers. All family caregivers reported life prolongation as a significant motivator for treatment, while the quality of life was rarely mentioned. A respectful relationship, close involvement, and open communication with healthcare professionals in the palliative setting were valued by many interviewees. Family caregivers’ experiences and needs seemed to be overlooked during medical encounters.

**Conclusions:**

Family caregivers of deceased patients with cancer mentioned life prolongation, and not quality of life, as the most important treatment aim. They highly valued interactions with the medical oncologist and being involved in the conversations. We advise medical oncologists to take more effort to involve the family caregiver, and more explicitly address quality of life in the consultations.

**Supplementary Information:**

The online version contains supplementary material available at 10.1186/s12904-021-00833-z.

## Background

Patients with incurable cancer, their relatives (i.e., family caregivers), and their healthcare professionals face complex treatment decisions [1–3]. During the treatment process, most patients value communication about treatment options and decisions with their family caregivers, as this affects the patients themselves, their decisions, and preferences [4–8]. Involving family caregivers also reduces patient distress and increases patient satisfaction, quality of life, autonomy, and treatment adherence [1, 9].

Thus, family caregivers are viewed as irreplaceable sources of support for patients with incurable cancer and play an important role in patients’ disease management and shared decision making [1, 4–6]. This latter concept is defined as a process between patients and their physicians, and if applicable, the patients’ relatives, whereby all parties share information, express their treatment preferences, and make a decision by mutual agreement [10–12]. Involvement of family caregivers in the shared decision making process is essential as the process and its outcomes also affect the family caregivers: it increases their quality of life and coping abilities with home-based palliative care [7, 8, 13, 14]. Moreover, to be able to support their relatives, family caregivers themselves need support and acknowledgment of their values and needs in the palliative phase [3, 15].

Despite the desire of the majority of family caregivers to participate to some extent in shared decision making [6, 8], a low level of involvement of family caregivers in palliative cancer care has been reported [4, 5, 13, 14, 16]. Additionally, the form and extent of family caregivers’ involvement can vary widely; from no involvement to dominance, from a direct to indirect influence (e.g., giving opinions about the treatments or the influence of the family member ‘just being present’), and from a positive to negative influence (i.e., the patients’, family caregivers’ and physicians’ appreciation of the family caregiver involvement in the decision making process) [8]. Whilst the importance of involving family caregivers in the shared decision making process of incurable cancer is acknowledged, clear insights into their experiences remain largely unexplained. Therefore, this qualitative interview study explored how bereaved family caregivers experienced the shared decision making process between their relative, themselves, and the medical oncologist.

## Methods

### Design and participants

Given its explorative nature, we performed a qualitative study between February and July 2020 as part of the CONtext project (Table 1). Medical oncologists and case managers purposively sampled family caregivers of their deceased patients with variability in family caregiver’s age, gender, and the patient’s cancer type. They included family caregivers aged 18 years or older of which the relative with cancer had died at least 6 weeks before the interview, and the deceased relative was treated while the CONtext project was running (2019-2020). Exclusion criteria were insufficient knowledge of the Dutch language and inability to answer interview questions. Family caregivers were approached by telephone by the medical oncologist or case manager of the deceased patient. During that phone call permission was asked whether the researcher (SO), experienced with qualitative research, could contact the potential participant. The researcher provided information and an information letter. After 1 week, the researcher contacted the family caregiver to confirm participation and to schedule the interview.

**Table 1:** The CONtext shared decision making process

Based on the shared decision making models of Elwyn et al. and van de Pol et al. [11, 12, 17] a quality improvement project, CONtext, was implemented at the Medical Oncology department of the Radboudumc in Nijmegen, the Netherlands. CONtext was integrated in five healthcare chains: gastroenterological oncology, gynaecological oncology, melanomas, urological oncology, and breast oncology. The project is based on collaboration between several departments: Centre for Oncology; Departments of Medical Oncology; Anaesthesiology, Pain and Palliative Medicine; Primary and Community Care; and Geriatrics.	
CONtext improves shared decision making for patients with incurable cancer should a decision be needed regarding their cancer treatment. This concerns moments when based on diagnostics (usually a CT scan) current therapy no longer seems to work or the disadvantages of current therapy no longer outweigh the benefits. CONtext explicitly focuses on the care process in the consultation room and offers patients opportunities to consciously think about their values and wishes within their contexts to deliberately decide about treatment. Moreover, it stimulates offering patients time-outs to discuss this with their general practitioner (GP) and their relatives. It also gives healthcare professionals tools to optimise this conversation with their patients.	
The implementation of the CONtext project included:	
1) Training medical oncologists (and fellows) and case managers in shared decision making and in applying the elements of the CONtext project. Almost all followed two small-group workshops and were invited to follow an eLearning course on shared decision making developed by, amongst others, the Dutch Federation of Cancer Patient Organizations. In addition, minimally two practical observations in the consultation room and group feedback sessions were organised by a member of the Person-centred Care Support team from the advisory group ‘Process Improvement and Innovation of the Radboudumc’. Moreover, the ‘Kantelkaart (Dutch)’, developed in the Connected Case Project of the Radboudumc was used to train healthcare professionals in discussing personal context and support patient in the decision making process. This card contained three questions for the patient to identify patient’s context and three reflection questions for the healthcare professional to support the patient. The patient questions were: 1. What is important to you right now? 2. What do you need for that? 3. How can I support you with that? The reflection questions for the healthcare professional were: 1. How can I ensure that the care for this patient is aligned with what he/she considers important? 2. What do I need for that? 3. Who can help me with that?	
2) Discussing the choices and treatment options considering the patient’s context, needs, wishes, and values in an outpatient consultation with the medical oncologist (in training) and case manager (*choice and option talk).* The medical oncologist (in training) focused on the medical context. After the outpatient consultation, the patient had a consultation with the case manager to further discuss the choices and treatment options focused on the wishes, values and needs of the patient in the patient’s context. Patients were prepared for shared decision making by explicitly mentioning the option of *shared decision making* and providing the ‘Kantelkaart’ to inventory personal context.	
3) Informing and inviting the GP (if the patient agrees) to remain actively involved in the patient’s care trajectory and help the patient with decision making.	
4) Striving for a two-week time-out period after the first consultation with the patient before making a medical decision. The patient could then consider and discuss the options with relatives or the GP. The case manager called the patient during these 2 weeks to offer support in the shared decision making process.	
5) Making the final decision about treatment in an outpatient consultation together with the medical oncologist (in training) and the case manager (*decision talk).*	
The CONtext shared decision making process is evaluated in, amongst others, a qualitative research project, where medical consultations are analysed and interviews with the involved medical oncologists, case managers, GPs, and patients are used. However, the family caregiver perspective remained unexplored, making the present research a valuable addition for the CONtext project evaluation.	
More information (in Dutch): www.qruxx.com/context	

### Data collection

In-depth interviews with the use of a topic list were conducted (Additional file 1). Topics were based on discussions by the research team, themes extracted from the literature, particularly from models about shared decision making [11, 12, 17], as well as initial implementation experiences of the CONtext methodology.

Due to the outbreak of coronavirus disease, telephone interviews instead of face-to-face interviews were performed. The interviews were conducted in Dutch from April to June 2020. The interviewer (SO) did not have any relationship with interviewees prior to the study. During and after each interview, field notes were made to isolate personal biases. At the end of the interview, demographic and medical information were collected using Castor EDC [18]. Data collection stopped when data were saturated.

Interviews were audiotaped, transcribed verbatim, and anonymised. Interview summaries, and a summary of the findings were sent to the family caregivers as member checks to ensure credibility [19], correct errors, clarify intentions, and provide additional information.

### Data analysis

Inductive content analysis, including constant comparison, was used for data analysis [20–23]. Analysis started after the first interview and resumed with each additional interview. The iterative process of data analysis and interview planning allowed the topic list to be updated regarding emerging themes, reflecting on preliminary results, and determining data saturation. Transcripts were continuously re-read, which also meant updating data categorisation.

Two researchers (SO and DE) independently coded the first three transcripts as closely as possible to family caregivers’ words to minimize subjectivity. Codes were compared and discussed until consensus was reached about a preliminary codebook, which was then used by the researcher (SO) to code the remaining transcripts. The codebook was updated after each interview (Additional file 2). If new codes emerged, all interviews were reviewed according to the new code. ATLAS.ti (version 8.4.20) was used to support the coding process. Open codes were combined into axial codes, categories, and themes, and discussed in research meetings until consensus was reached. The researcher (SO) translated the interview excerpts to English. The Consolidated criteria for reporting qualitative research (COREQ) were used for reporting [24]. Standard descriptive statistics were used to describe the quantitative data using IBM SPSS Statistics (version 25).

### Ethical approval

All methods were carried out following the relevant guidelines and regulations. Participants were not subject to treatment, nor were they required to behave in a particular way. Therefore, the Medical Review Ethics Committee region Arnhem-Nijmegen concluded that this study was not subject to the Medical Research Involving Human Subjects Act and approved the study (case number 2020-6105). All family caregivers gave verbal informed consent prior to the interviews, which were recorded on audiotape. The method of consent was approved by the Medical Review Ethics Committee region Arnhem-Nijmegen.

## Results

Table 2 shows the characteristics of the family caregivers and the deceased family members. All sixteen family caregivers participated (median: 69 min; 40-135 min) and all approved the interview summaries. In total, 285 codes were retrieved, grouped into 63 axial codes, nine categories, and four themes: 1. scenarios of decision making, 2. future death of the patient, 3. factors influencing choices when making a treatment decision, and 4. preconditions for the decision making process.

**Table 2:** Characteristics of the family caregivers (*n* = 16) and the deceased family members with incurable cancer^a^

Characteristic	Family caregivers		Deceased patients	
	Number (%)	Median (range)	Number (%)	Median (range)
Gender, female	7 (44)		10 (63)	
Age in years		68 (30-79)		69 (30-85)
Religion, yes	11 (69)		12 (75)	
Educational level			NA	
Primary education Secondary education Higher education	1 (6) 6 (38) 9 (56)			
Relationship with the deceased patient			NA	
Spouse/partner Child Brother in law	14 (88) 1 (6) 1 (6)			
Providing care for the patient, yes	15 (94)		NA	
Months as caregiver		10 (0.3-72)		
Days since patient’s death		187 (40-331)	NA	
Primary cancer diagnosis	NA			
Gynaecological Gastrointestinal Melanoma Sarcoma Urological Squamous cell carcinoma of unknown origin			6 (38) 3 (19) 2 (13) 2 (13) 2 (13) 1 (6)	
Place of death	NA			
At home Institution Hospice Hospital Recovery centre/rehabilitation home			10 (63) 6 (38) 4 (25) 1 (6) 1 (6)	
Euthanasia, yes	NA		2 (13)	

*NA* not applicable

^a^The age of the deceased patient, cancer diagnosis, and, if noted, the age of the family caregiver and date of death were verified by a medical oncologist in the electronic health records of the deceased patient

### Theme 1: scenarios of decision making

Four scenarios of decision making were addressed by the family caregivers in which the person(s) involved in making treatment decisions varied: 1) shared, 2) patient decides, 3) caregiver decides, and 4) physician decides. First, few family caregivers experienced the decision making process as being either collaborative or in mutual agreement with the physician, patient and family caregiver: *‘Because this is how my wife and I saw it, we both [patient and family caregiver] saw it this way. It was almost like a kind of teamwork between the patient and the physician.’ (FC001).* Often in the case of long-married couples, the family caregiver felt equally involved by the patient in decision making. *‘Everyone makes their own choices. And we [the patient and family caregiver] made our choices together, and as I said, we were on the same page and sometimes we just had to look at each other (..), that was enough for us to know.’ (FC011).*

Second, most interviewees stated that final decision making was done by the patient, which was perceived as the most desirable situation because treatment choices had consequences for the patient’s life and body. In this scenario, family caregivers provided emotional and informational support, participated in conversations with physicians, gave advice, and had a protective role towards the patient. Family caregivers’ motivations for being supportive were feelings of love, respect, and responsibility. *‘Interviewer: why did you stand behind him [the patient] like that?’ ‘FC008: Out of love. Also, to take care of him. But you know, if your partner gets seriously ill, everything also changes in a relationship. And he, he went through it [the treatments]. Not me. So, then you literally are standing on the side-line; I was both beside him and behind him.’ (FC008).*

Third, a minority of family caregivers expressed that they dominated the decision making, especially if patients were experiencing difficulties making choices themselves. *‘My wife has never been much of a talker and making decisions was very difficult for her. She followed my lead in all decisions, which was quite difficult for me, because I had the feeling that I was making vital decisions for her.’ (FC003).*

Fourth, more than half of the family caregivers and patients deferred decisions to their physicians. *‘We [the patient and family caregiver] agreed quite quickly; we took their advice [the physicians]… they are our experts. We listen to what they say and, from that, we choose what we need to choose.’ (FC005)*. However, when medical oncologists decided to stop life-prolonging treatment, not all family caregivers and patients agreed with the physician.

In all these scenarios, family caregivers identified several essential steps before making treatment choices. They indicated that discussing all the treatment options and potential results with physicians was important. *‘It was all well explained [the treatment options]. I have to say that, also when either the oncologist or internist or gynaecologist or whoever said, ‘We can do it this way or that way (..). These are the options.’ We discussed all those options with the medical staff. What they [the physicians] did was just to offer choices (..). You have to make the decision yourself, but with compelling advice [of the physicians].’ (FC014).* Consideration of the patient’s and family caregiver’s opinions and preferences was also valued in conversations with physicians, as well as seeking advice from other healthcare professionals and other family members. *‘I experienced that the decisions were made (..) taken into account the viewpoint of the patient and the viewpoint of the environment, which was just me (..), and based on that an advice was given. They [the physicians] expressed: “we would like to do this or how would you [the patient and family caregiver] feel about that, what do you [the family caregiver] think?”’ (FC014).* In almost all cases, treatment choices were first discussed between the family caregiver and patient before deciding. *‘Then [in the car] we [the patient and family caregiver] always looked back on the conversation, asking: What did you think? What did you think of what the oncologist said, or what did you think of the oncologist, because sometimes we had several [oncologists] of course. And now, we discussed what will we do... things like that. It [the conversation] went back and forth; we were on an equal level too.’ (FC008).* Some family caregivers explicitly indicated that treatment choices were made rationally and realistically: exploring all options, weighing pros and cons, and choosing the best option in their opinion.

GPs and palliative care teams in the hospital had a marginal role in cancer treatment decision making; according to some family caregivers, any decision making involvement was undesirable. *‘No, no. (..) Because he [the GP] knows a bit about everything, but he’s not a specialist in cancer or other diseases. (..) The GP helped with practical things [arranging an ambulance], but not with substantive things about the disease.’ (FC002).* When end-of-life decisions had to be made, the GP and palliative care team had a more prominent role.

### Theme 2: future death of the patient

According to family caregivers, physicians prepared the patients and family caregivers for the patient’s future death by saying ‘Mr *name*, we have very bad news. The MRI shows this, this and this. You only have a very short time left to live. Do you hear what I say? You only have a very short life left to you.’ (FC008). Often, this did not surprise the family caregiver. *‘I would have been very surprised if she could have been cured. Maybe I was pessimistic, but maybe realistic. I thought, this isn’t going to work, no.’ (FC010).*

Many family caregivers appreciated discussing death, the dying process, and the prognosis with the physicians. *‘I think that if physicians discuss everything with the patients, including death…. I think that’s what they [physicians] did. We talked quite a lot about death ourselves, then that is very comforting. (..) Having a conversation on these subjects is really important, because that is what awaits you.’ (FC005).* However, for some family caregivers and/or the patients, discussing death and end-of-life choices was not (yet) open to discussion. *‘He [the medical oncologist] was a nice man, but he said… and we [the patient and family caregiver] never use that word… “you’ll die”. That wasn’t exactly tactical.’ (FC001).* Sometimes, the patient’s terminal condition had not (yet) been acknowledged, and people’s emotions increased when talking about death. *‘He [the patient] did not want to talk about death or about the fact that he could seriously… that his condition could deteriorate. He said, ‘I’m getting better, I’m going to be the medical wonder.’ (..) He decided, ‘I’m going to survive’, and didn’t want to hear anything else.’ (FC008).*

The infaust prognosis was still ‘a slap in the face’ for most family caregivers. The use of the words ‘death’ and ‘palliative’ confronted approximately half of the family caregivers. *‘When I heard “palliative”, I already thought, oh dear, that has to do with dying. But that [palliative care] was also more about spiritual assistance when going through this process. Anyway, that was, in fact, the beginning of the end.’ (FC012).* Sometimes, the medical oncologist gave an estimation of a patient’s life expectancy. This estimation was not always correct, which could be experienced as burdensome by the family caregiver.

All family caregivers perceived the disease process as burdensome, especially in the case of fast progression. After the patient’s death, many family caregivers emotionally went through a reflective period with ‘what if’ scenarios. *‘Sometimes I still have the question, but I really don’t want to get stuck on that, but would it have helped if we had not had to wait six, seven weeks, but could have had surgery six, seven weeks earlier?’ (FC003).* This scenario thinking was acknowledged to be meaningless as it would not bring the patient back to life.

### Theme 3: factors influencing choices when making a treatment decision

All bereaved family caregivers reported the possibility of extending the patient’s life as the main reason to opt for treatment. Interviewees mentioned the patient’s urge to keep going and not to give up on life. *‘My wife and I were also very committed to being together as long as possible. Look, if you are faced with that choice, if we start chemo and it’s effective, you could have a few more years, so to speak. Hopefully as long as possible, but at least a few more years.’ (FC003).* Anticipated regret about refusing a potentially beneficial treatment and naturally clutching at any semblance of hope were important motivations to pursue each offered treatment option. *‘If he [the patient] had not done anything [treatments] from the beginning, we would have… then we would have said, it will be about three months, maybe six. But that was more than a year and a half ago and that’s why… That’s what he [the patient] meant by “doing everything to stay alive”.’ (FC009).* Moreover, most family caregivers had the perception that there was only one ‘good’ treatment option. ‘It wasn’t that there was a range of options we could choose from. *laughs* The options for choice were quite limited.’ (FC010). It felt like there was no choice. *‘I said, Mom, are you sure you want this [the treatment]? Then she said, Yes. I don’t want to die. There’s no other option, so I’ll do it.’ (FC004).*

Few family caregivers stated maintaining a patient’s quality of life as an important factor in treatment decisions. *‘Then you stop the immunotherapy [if it makes you very ill]. Then he [the patient] wouldn’t have to choose to be ill. That wasn’t his choice, to still have that next immune session. No, none of that was relevant, no, because as I said, in everything he’s done, quality of life has always been his motivation.’ (FC005).* All the other family caregivers did not mention quality of life as treatment aim.

Family caregivers and patients also considered the pros and cons of the treatment. Chances of improvement made choices easier for some family caregivers. *‘We knew that immunotherapy had a small chance of success, but it was an opportunity. So, I didn’t think that [immunotherapy] was too bad.’ (FC005).* In contrast, if the chance of treatment success was small, some other family caregivers expressed treatment as ‘not worth it’. The risk of side effects and complications was also mentioned: *‘She wouldn’t choose that, because the side effects were so bad that she would deteriorate physically, and then it would actually be better if she didn’t do it, and have a…well… a restful phase of life, final phase, so to speak.’ (FC001).* Sometimes, patients decided to continue treatment despite the side effects.

Moreover, the development of complications or worsening of the patient’s condition influenced treatment choices: often to (temporarily) stop treatment. Some family caregivers reported that, at that point, the patient accepted his/her situation and approaching death. *‘There was no more saving… that intestinal perforation and then the metastases in the back and those in the legs (..), and after a few peritoneal lavages, he [the patient] said, “I don’t want to live anymore, I can’t be cured. It’s good for me.” And he was at peace with that.’ (FC011).* In contrast, a few interviewees mentioned that their deceased relative could not accept the terminal condition and continued to believe they could get better. As a result, these patients kept pushing their boundaries and chose to continue treatment till death.

### Theme 4: preconditions for the decision making process

Family caregivers experienced three preconditions for decision making. A first precondition is a suitable way for physicians to approach the family caregivers. All family caregivers described the importance of the physicians’ respectful approach. *‘Particularly, the respect for the person in front of you - the patient. (..) That’s something that’s incredibly important for any healthcare professional to constantly monitor; who am I [the physician] facing? That it [the care] does not become standard.’ (FC013).* Other family caregivers mentioned close involvement; a good relationship; good listening; and empathetic, human interaction. *‘My wife was actually taken very seriously and treated specially. She always said, “I’ve been treated like a princess, I’ve been treated like a queen.”’ (FC001).* Some family caregivers explicitly addressed the importance of a personal approach. *‘That you [the patient] feel that the person who is treating you [the physician] is also involved, and that you’re a patient with your own identity and not a number. That you’re receiving personal treatment and that the person giving the treatment also has a human face, and is not a robot.’ (FC015).* Remarkably, almost all family caregivers mentioned an unpleasant approach by some physicians during treatment. *‘That they [the physicians] very often looked away, like, “ah, that’s a young couple, we won’t take it seriously”. We were often treated like children rather than adults.’ (FC016).* Specifically, this entailed not listening carefully, a factual and distant way of contact, and little involvement. *‘It was all about, “When do we make the choice for chemotherapy and when can we start it?” So, it was kind of, inhuman, let’s put it another way, it was a bit distant.’ (FC006).* Family caregivers mentioned that these experiences could eventually lead to decreased trust.

A second precondition is the physicians’ way of communication. Physicians’ honest, clear and open communication could facilitate family caregivers’ involvement in decision making. *‘At least warmth. At least time. Having all the time. Open and honest, that you have the feeling, it can happen to all of us. (..) And that there’s always a listening ear, someone you can always call and that it’s never too much.’ (FC013).* Some family caregivers mentioned appreciating physicians’ reassuring wording and their ability to give hope. *‘We [the patient and family caregiver] can never talk about it [the cancer] like the physicians do. They seem to make it less awful, let me put it that way, mitigating the circumstances a bit, you know. They know exactly how to deal with a patient.’ (FC007).* However, most family caregivers noted some absence of communication with (and between) physicians and discontinuity in medical personnel. *‘That suddenly there was another physician who actually, well, had to bring bad news for example, that we [the patient and family caregiver] had not expected. (..) The fact that suddenly someone unexpected is sitting opposite you, that’s the moment it went wrong. So, at those moments, communication could actually have been better.’ (FC013).*

The third precondition for decision making is physicians’ alignment to the family caregivers’ personal needs for attention and guidance. Some family caregivers were satisfied with the attention (or lack thereof) they received. *‘I think they [the physicians] took an incredible amount of time, each time, and paid a lot of attention to the situation we [the patient and family caregiver] were in. They did well.’ (FC005).* While others were not: some family caregivers noted that physicians should have empathised better with their needs in taking care of their family member*. ‘I think that the partner sitting next to the patient can sometimes be included a bit more (..), my husband said “no” [to discussing death and end-of-life choices] every time, but I said “yes”, and then they [the physicians] could have gone a bit more to the “yes”. (..) Maybe they [the physicians] should have taken me aside in a room to say “listen, this is how it is and how are you doing?”.’ (FC008).* Other family caregivers wanted less attention for themselves: ‘I would have preferred that she [the physician] had not asked me at all, so to speak. (..) because my wife was my center of attention. *cries* But yes, everyone is different, you know.’ (FC007).

## Discussion

### Main findings

We explored the experiences of bereaved family caregivers with the shared decision making process that had taken place between their relative, themselves, and the medical oncologist. Most family caregivers deferred final decision making to the patient or physician. Family caregivers mentioned life prolongation as the most important motivator for treatment in the incurable cancer setting, while the quality of life was rarely mentioned. Moreover, family caregivers’ needs and experiences seemed to be overlooked during medical encounters. They valued being seen by the medical oncologist and being involved in the conversations about treatment decisions.

### Strengths and limitations

A strength of this study is the in-depth insights into the experiences of bereaved family caregivers with shared decision making. Physicians can now take family caregivers’ personal experiences into account, connect and align with these experiences, and understand what the family caregivers are going through in the palliative stage. Moreover, family caregivers were interviewed after their family member died, therefore, they were able to reflect on all the decisions made in the palliative phase. There are also some limitations. Recall problems cannot be precluded. As family caregivers were interviewed within months after their family member had passed away, their grief and the length of time since the death might also have influenced their memories of the shared decision making process. Kahneman showed with his peak-end rule theory that memory of a certain period or event is influenced by its peaks and end [25, 26]. Moreover, it should be named that sampling was based on healthcare professionals’ considerations, and the sample mainly included highly educated partners. Another point is that we interviewed family caregivers from patients treated at a Dutch tertiary university hospital. Presumably, our results are transferable to similar settings: developed countries where patients with incurable cancer are still treated in hospitals and where autonomy and attention to personal wishes are highly valued in palliative care [27]. Results may differ in other settings and for family caregivers with a different relationship to the deceased. Therefore, future research will benefit from studies with more diverse populations.

### What this study adds

Striving for a patient’s life prolongation was found to be an important aspect influencing treatment choices. Previous studies on treatment decisions in advanced cancer care also report possible life prolongation as the main reason to opt for treatment [28, 29]. Moreover, our study shows that extending a patient’s life as a motivator is almost self-evident, where family caregivers and patients naturally clutch at any semblance of hope and accept each treatment option. The strong focus on life prolongation may have been influenced by the close (mostly spousal) relations and the desire to gain more lifetime together [4, 30], or the academic setting. Another explanation might be that family caregivers and patients lack sufficient information about the decision to withhold or withdraw treatments and decide for supportive care or ‘doing nothing’ [3].

Palliative care aims to maintain optimal quality of life, which has been reported as an influencing choice factor for palliative cancer treatment [15, 28]. Even though stopping therapy at some point might be the best way to preserve quality of life, only a few family caregivers in our study stated that the patient had opted to stop treatment. Striving for quality of life becomes significant only when life prolongation is no longer possible. We cannot determine whether physicians experienced difficulties in discussing quality of life aspects, or if patients’ and family caregivers’ understanding was suboptimal. Literature does suggest that physicians in advanced cancer care do not discuss the available treatment options in an equal way and that patient’s awareness of all the treatment options is limited. Physicians barely address and integrate the patients’ context, i.e., their values, goals, and wishes in decision making, as they tend to focus on the medical aspects [31–33]. At the very least, in the shared decision making process it is necessary for physicians to explicitly discuss (dis)advantages of quality of life aspects of withholding treatments, and verify family caregivers’ and patients’ understanding.

Focus on life prolongation and physical treatment may explain that family caregivers were satisfied with the marginal role of the GP through the whole process of a patient’s illness. With the GP only involved in the terminal phase, it seems that palliative care has not yet become an integral part of cancer care as proposed by the World Health Organization [34]. GPs experience difficulties in initiating advance care planning discussions with patients still being treated in hospitals, as these patients are often not open to discussion [35]. Our study confirms this from the family caregiver’s perspective.

We found that many family caregivers missed receiving attention to their own needs and felt they were not actively involved in the shared decision making process. Only a few interviewees experienced the shared decision making process as a mutual agreement between physician, patient, and family caregiver [10]. Family caregivers’ feelings of being unprepared for the palliative phase can be burdensome [13, 14], and physicians tend to underestimate family caregivers’ need for information about death, dying, and palliative care [36]. A significant reciprocal relationship between the patient’s and the family caregiver’s distress is also acknowledged [37]. This suggests that the patient-caregiver dyad reacts as an ‘emotional system’, influencing each other, and that they should be viewed as one unit. In our study, this ‘emotional system’ is implied through family caregivers’ raised emotions when talking about their family member having pain or distress, and their descriptions of the loving, close relationship with their family member. Early palliative care that also focuses on the family caregiver and, if applicable, refers the family caregiver for counselling to another healthcare professional seems a promising way to reduce family caregivers’ distress and meet their personal needs [38, 39]. The family GP may be the most appropriate professional for this task. Future research should explore implementing this in clinical practice.

Finally, our results confirm that family caregivers’ experiences vary widely [14]. Some family caregivers wanted to talk about the patient’s approaching death and wanted attention for themselves in the shared decision making process, while others did not. In line with other studies [3, 13, 40, 41], because of family caregivers’ diverse experiences and preferences, our study emphasises the importance of open person-centred communication and a personal relation with healthcare professionals, and listening to and respecting both the patient and family caregiver. Consequently, we advise physicians to take the patient’s and family caregiver’s wishes into account and discuss their preferred roles in the shared decision making process.

## Conclusions

Our study highlights that family caregivers value being seen by the medical oncologist and being involved in the conversations, even though family caregivers do not perceive a large role for themselves in final decision making, as they often defer decisions to the patient or the physician. Therefore, clinical strategies to assist physicians to practically engage with family caregivers and systematically assess their experiences and needs while not undermining the patient focus are needed. Findings from our study identify life prolongation as being the most important treatment aim in the palliative phase. In contrast, preserving the quality of life was rarely mentioned by family caregivers. We advise medical oncologists to explicitly address and discuss quality of life aspects in the consultations and verify family caregivers’ and patients’ understanding of the process.

## Supplementary Information

**Additional file 1.** Topic list family caregivers. Microsoft Word document (DOC). In this additional file the topic list for the interviews with the family caregivers is reported.

**Additional file 2.** Codebook interviews family caregivers. Microsoft Word document (DOC). In this additional file the final coding scheme for the interviews with the family caregivers is reported.

## Data Availability

The data supporting the findings of this study are not publicly available due to individual privacy, but are available from the corresponding author on reasonable request.

## Abbreviations

GP: General practitioner
