# Supplementary material for: Experiences of bereaved family caregivers with shared decision making in palliative cancer treatment: a qualitative interview study
Source: BMC Palliat Care. 2021 Sep 7;20:137. doi: 10.1186/s12904-021-00833-z (PMC8423331; doi:10.1186/s12904-021-00833-z)
Supplement: Supplementary file 1 — Additional file 1. Topic list family caregivers. Microsoft Word document (DOC). In this additional file the topic list for the interviews with the family caregivers is reported. [file 12904_2021_833_MOESM1_ESM.docx]

**Additional file 1.** Topic list family caregivers

| *Ideally, nondirective, open-ended questions will be asked. All questions will be adjusted per family caregiver by using the relationship with the deceased loved one (your mother, wife, etc..) to make it recognisable.*  1) How did the disease process of your deceased loved one go? *(this question is only asked to gather some specific background information on the disease process, which is also explained to the interviewee)*  *-* What have been the most important decisions for you regarding the treatment of your deceased loved one?  2) What are your experiences with the decision making process regarding treatment of your deceased loved one? How did you experience making choices about treatment options?  - How did the decision making process about treatment X go?  *-* When was the choice made about treatment? How much time did you have to make choices?  - To what extent was the decision making ‘shared’? *(if applicable, explain the term ‘shared’: patient and physician are both involved in the decision making process, whereby they both share information, express their treatment preferences and make a decision by mutual agreement).*  - What did you experience as pleasant in the decision making process? And what did you experience as less pleasant?  3) How did you experience your role in the decision making process?  - How much were you involved in the decision making process?  - Was this different compared to earlier decisions about treatment?  - What did you think/feel about the extent to which you were involved in decision making?  - What/who made that you were involved in this way?  4) What were your expectations about the decision making process?  - How was that compared to how it finally went?  - What are the points for improvement in this decision making process?  5) What was the role of the general practitioner (GP) in the decision making process regarding the treatment of your deceased loved one?  - When did the GP play this role in the decision making process?  - How satisfied are you with this role of the GP?  - How would you prefer the role of the GP during the decision making process?  6) Did the patient have a (specialised) nurse/case manager?  - If so: What was the case manager’s role in the decision making process regarding the treatment of your deceased loved one?  - When did the case manager play this role in the decision making process?  - How satisfied are you with this role of the case manager?  - How would you prefer the role of the case manager during the decision making  process?  7) What were you most concerned about during the decision making process regarding the treatment of your deceased loved one?  - Did the healthcare professionals in the Radboud university medical center (Radboudumc) pay attention to what you might have worried about, and what you were most concerned about?  8) What do you already know about the ‘CONtext’ project at the Radboudumc? *(possibly give some information: CONtext is a quality improvement project, since the beginning of 2019 to improve decisions on treatment options for patients with cancer in the physician’s office in the Radboudumc. Interaction and connection between patient, GP and hospital is central.)*  - If so: From whom did you hear that?  - What have you noticed about the ‘CONtext’ project? |
| --- |
