# Supplementary material for: Experiences of bereaved family caregivers with shared decision making in palliative cancer treatment: a qualitative interview study
Source: BMC Palliat Care. 2021 Sep 7;20:137. doi: 10.1186/s12904-021-00833-z (PMC8423331; doi:10.1186/s12904-021-00833-z)
Supplement: Supplementary file 2 — Additional file 2. Codebook interviews family caregivers. Microsoft Word document (DOC). In this additional file the final coding scheme for the interviews with the family caregivers is reported. [file 12904_2021_833_MOESM2_ESM.docx]

**Additional file 2.** Codebook interviews family caregivers

| **Themes** | **Categories and axial codes^1^** |
| --- | --- |
| 1. Scenarios of decision making | 1. Who makes the treatment choice(s)?   - The physician makes the treatment choice - The family caregiver and patient disagreed with the physician’s choice of treatment - The family caregiver makes the treatment choice - The patient makes the treatment choice - The family caregiver agrees to the patient’s treatment choice and puts the patients first - Decision making was together, shared decision making - God decides on life and death - The case manager(s) does not contribute to making a treatment choice - The general practitioner (GP) does not contribute to making a treatment choice - The GP is involved in end-of-life decision making - The palliative care team is or is not involved in decision making   2. Additional role of the family caregiver during the decision making process and disease process^2^   - The family caregiver supports the patient - The family caregiver is a spokesperson - The motivation of the family caregiver to be involved in the decision making process the way he/she did - The family caregiver searches for information about the disease and treatments - The family caregiver is protective towards the patient   3. Actions in the decision making process   - Keep thinking rationally and realistically at decision moments - The patient and family caregiver are advised on the treatment choice - The patient and family caregiver discuss treatment options together - The patient and family caregiver indicate preferences - Taking time to make treatment choices - The physician discusses treatment options with the patient and family caregiver |
| 2. Future death of the patient | 4. The patient and family caregiver are talking about death and dying   - The patient and family caregiver discuss life expectancy with physicians - Death could be discussed with the patient, family caregiver and physician - Death could not yet be discussed with the patient, family caregiver and physician - Awareness of the patient and family caregiver about the poor prognosis - Denial of possible bad result/assuming positive results   5. Fears and concerns of the family caregiver and patient during the disease process   - Fears and concerns for the future and further course: the patient’s death   6. Fears and concerns of the family caregiver after patient’s death (in retrospect)   - Patient’s death is/was burdensome - Decisional regret: The family caregiver describes ‘what if’ scenarios |
| 3. Factors influencing choices when making a treatment decision | - Strong motivation to undergo treatments: There is no other choice - Life extension, not wanting to die - Quality of life and quality of dying - Personal values and norms of the patient and family caregiver - Practical factors - Intuition/feelings - Deterioration of patient’s condition - Acceptance of patient’s condition and the disease process - Risk of complications/side effects - Chance of a curation - External references (opinion and information of others) - Uncertainty/unfamiliarity about treatment option - Time pressure (wanting a treatment as soon as possible) |
| 4. Preconditions for the decision making process | 7. Negative points of care and the care personnel^2^   - Treatment, attitude and approach of the physician towards the patient and family caregiver - The physicians paid little attention to the family caregiver - The physicians paid too much attention to the family caregiver - Practical factors - Uncertainty, indistinctness about care - Absence of care and communication (also communication between healthcare professionals) - The family caregiver is dissatisfied with the advice and statements of the physician - Dissatisfaction with the pace of care   8. Positive points of care and the care personnel^2^   - Family caregiver’s satisfaction with the patient’s care and healthcare professionals in general - Treatment, attitude and approach of the physician towards the patient and family caregiver - Personal approach - Practical factors - Satisfaction with the pace of care - Possibility for a contact point and contact with healthcare professionals - Way of communicating - Medical expertise - Continuity in medical personnel and communication within healthcare professionals - Physicians paying attention to the patient and family caregiver   9. Relationship between the family caregiver and the patient   - The family caregiver and patient have an intimate, equal relationship - The family caregiver and patient have a less intimate, equal relationship |

^1^Open coding results are on request available. Not all axial codes are elaborated in the results section of the present study.

^2^ It was difficult for family caregivers to distinguish between the decision making process and the disease process in general (for the topics: role, and barriers and facilitators). Therefore, these categories include both the period of the decision making process and the disease process in general.
